# A novel machine-learning aided platform for rapid detection of urine ESBLs and carbapenemases: URECA-LAMP

**DOI:** 10.1128/jcm.00869-24

**Published:** 2024-10-24

**Authors:** L. Ricardo Castellanos, Ryan Chaffee, Hitendra Kumar, Biniyam Kahsay Mezgebo, Pawulos Kassau, Gisele Peirano, Johann D. D. Pitout, Keekyoung Kim, Dylan R. Pillai

**Affiliations:** 1 Department of Pathology & Laboratory Medicine, Medicine, and Microbiology, Immunology and Infectious Diseases, University of Calgary, Calgary, Alberta, Canada; 2 Department of Mechanical and Manufacturing Engineering, University of Calgary, Calgary, Alberta, Canada; 3 Department of Biosciences and Biomedical Engineering, Indian Institute of Technology Indore (IIT Indore), Indore, Madhya Pradesh, India; 4 Amhara Public Health Institute, Amhara Bahir Dar, Ethiopia; 5 Alberta Precision Laboratories, Calgary, Alberta, Canada; Johns Hopkins University, Baltimore, Maryland, USA

**Keywords:** antimicrobial resistance, point-of-care testing, POCT, near-patient testing, loop-mediated isothermal amplification, diagnostic tests, machine learning, UTI

## Abstract

Pathogenic gram-negative bacteria frequently carry genes encoding extended-spectrum beta-lactamases (ESBL) and/or carbapenemases. Of great concern are carbapenem resistant *Escherichia coli*, *Klebsiella pneumoniae*, *Pseudomonas aeruginosa,* and *Acinetobacter baumannii*. Despite the need for rapid AMR diagnostics globally, current molecular detection methods often require expensive equipment and trained personnel. Here, we present a novel machine-learning-aided platform for the rapid detection of ESBLs and carbapenemases using Loop-mediated isothermal Amplification (LAMP). The platform consists of (i) an affordable device for sample lysis, LAMP amplification, and visual fluorometric detection; (ii) a LAMP screening panel to detect the most common ESBL and carbapenemase genes; and (iii) a smartphone application for automated interpretation of results. Validation studies on clinical isolates and urine samples demonstrated percent positive and negative agreements above 95% for all targets. Accuracy, precision, and recall values of the machine learning model deployed in the smartphone application were all above 92%. Providing a simplified workflow, minimal operation training, and results in less than an hour, this study demonstrated the platform’s feasibility for near-patient testing in resource-limited settings.

**IMPORTANCE:**

Extended-spectrum beta-lactamases (ESBL) and carbapenemases confer resistance to third-generation cephalosporins and carbapenems in pathogenic Gram-negative bacteria such as *Escherichia coli*, *Klebsiella pneumoniae*, *Pseudomonas aeruginosa,* and *Acinetobacter baumannii*. Conventional antimicrobial susceptibility testing is based on phenotypic methods, and results can take several days to be obtained. Current genotypic detection methods can be rapid but require expensive equipment and trained personnel. In this study, we present a novel machine learning-aided platform for the rapid detection of ESBLs and carbapenemases using Loop-mediated isothermal Amplification (LAMP). The validation of the platform demonstrated percent positive and negative agreements above 95% for all targets. The newly developed platform provided a simplified workflow, minimal technical training, and results in less than an hour. This study demonstrated the platform’s feasibility for rapid testing of ESBL and carbapenemases in bacteria and urine specimens.

## INTRODUCTION

Antimicrobial resistance (AMR) is a major global health problem (1). Of particular interest is resistance conferred by carbapenemases and extended-spectrum beta-lactamases (ESBLs) in Gram-negative bacteria (2–4). Carbapenemase and/or ESBL-producing Gram-negative bacteria are considered critical priority level for research and development of new antibiotics by the World Health Organization (WHO). This prioritization is based on the availability of effective therapy, overall mortality, burden to health care systems, and increasing drug resistance (4). Among Gram-negative bacteria, carbapenem-resistant *Escherichia coli*, *Klebsiella pneumoniae*, *Pseudomonas aeruginosa*, and *Acinetobacter baumannii* were estimated as top causative agents of deaths in 2019 attributable to and associated with AMR worldwide. These bacteria were responsible for close to a million global deaths in the same year (1).

Urinary tract infections (UTIs) are among the most common infections caused by Gram-negative bacteria (5, 6). In a previous report, at least 10%–15% of UTI in patients that visited the emergency department were associated with urosepsis (7). Enterobacterales such as *E. coli* and *K. pneumoniae* and non-lactose fermenting *P. aeruginosa* and *A. baumannii* are a common source of carbapenem-resistant UTI in healthcare settings (8, 9). An increased healthcare burden is associated with the presence of carbapenem-resistant pathogens in UTI patients, especially when infections are complicated by bacteremia (8). Early identification of patients with high risk of bacteremia and/or carbapenem-resistant UTI is needed to reduce this burden.

The lack of appropriate AMR diagnosis continues to be one of the drivers for unsubstantiated use of antimicrobials in both high and low-middle income countries (10, 11). Conventional antimicrobial susceptibility testing during infections is based on phenotypic methods. These methods can only be performed once a pure culture of a microorganism is obtained and can take several days to provide results (12). Molecular methods such as PCR and qPCR have been developed for the detection of emerging pathogens and associated AMR (13). However, these methodologies often require expensive equipment, reagents, and trained personnel. These prerequsites are often absent in resource-limited and remote settings (14). The need for rapid diagnostics to help tackle the global problem of AMR has been extensively underscored (2, 3, 15, 16). Access to affordable diagnostic tools, including those which are point-of-care (POC), is recommended for effective implementation of national and global action plans to tackle AMR (16). Loop-mediated isothermal amplification (LAMP) is a rapid alternative to PCR. LAMP was originally reported in 2000 by Notomi and colleagues and has become a well-established method for the detection of infectious agents (17, 18). Several LAMP assays have been developed into commercial kits and/or adopted as recommended methods for routine identification and surveillance of pathogens (18–20). LAMP takes place under isothermal conditions which a simple heat block can provide. It is, thus, independent from the use of specialized instruments like PCR thermocyclers, making it a cost-effective technique. DNA extraction can be prepared from a large variety of sample types, facilitating detection from crude extracts without the need for DNA purification (14). For these reasons, LAMP has the potential to be integrated into POC testing and near-patient testing (NPT) methodologies (21).

Despite its advantages, LAMP has some limitations. First, commercial LAMP assays are expensive and mainly affordable in high-income countries (22). Second, the measurement of LAMP products relies mostly on indirect analysis, like turbidity and non-specific dyes, and may require post-amplification manipulation, which can lead to possible cross-contamination or reduced specificity (23). To overcome these limitations and building on previous experience in our group (24), we developed the URine, ESBL, and CArbapenemase (URECA)-LAMP platform. The platform contains a low-cost all-in-one device for DNA extraction, LAMP amplification, and results visualization. It also contains a detection panel for the most prevalent ESBLs and Carbapenemases and a machine-learning-based smartphone application for automated interpretation of results. The (URECA)-LAMP platform was designed to leverage the NPT potential of LAMP-based screening of ESBL and carbapenemases in clinical isolates and urinary infections.

The aim of the present study was to develop and validate the URECA-LAMP platform for rapid and cost-effective NPT screening of the most relevant ESBL and carbapenemase genes in critical priority Gram-negative bacteria. Our results indicate the URECA-LAMP platform has the potential to be successfully implemented in the clinical setting.

## MATERIALS AND METHODS

### Novel device for performing LAMP assays

**Fig 1:**
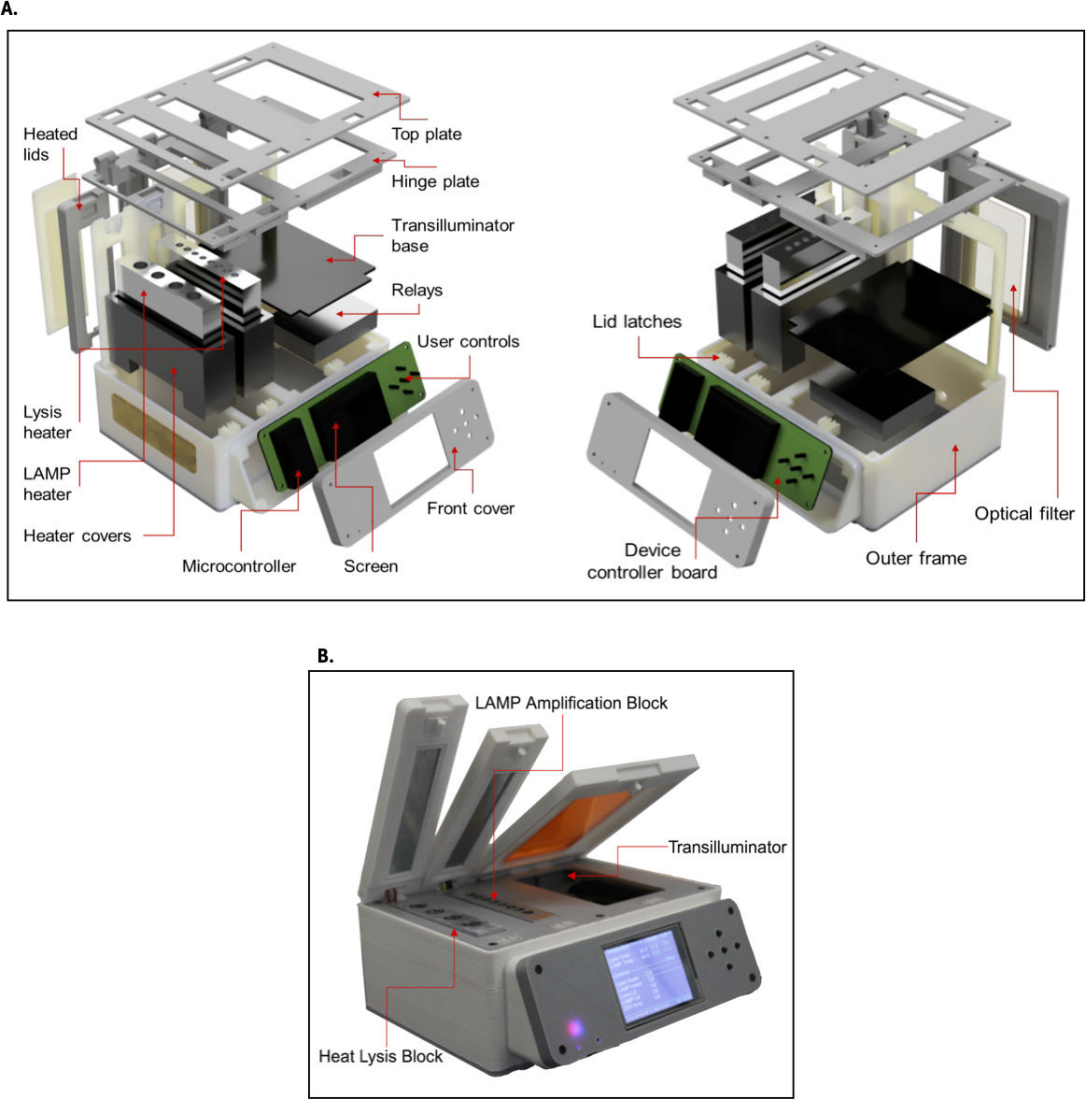
WHITE PEARL construction scheme. (**A**) Internal design and components. (**B**) Manufactured prototype with the three primary modules for performing LAMP assays.

To perform the sequence of steps in the URECA-LAMP assay, we developed a custom-made device coined “White Pearl.” The steps consist of bacterial lysis, isothermal incubation, and visual fluorescent detection. To facilitate ease of usage without the requirement of technical expertise and training, the device was simplified to remove steps of centrifugation for DNA extraction (24). The 3D design for the White Pearl device was prepared using Autodesk Fusion 360 (Autodesk, CA, USA). All the structural parts of the device were fabricated using polycarbonate and poly-lactic acid (PLA) materials and a fused deposition modeling (FDM) 3D printer (Anycubic C, Commerce, USA). Appropriate thermal insulation materials were used wherever required. The White Pearl device consisted of three primary modules directly involved in the assay workflow—(i) heating block for thermal lysis maintained at 95°C, (ii) heating block for isothermal amplification maintained at 64°C, and (iii) a transilluminator for visual detection of fluorescent readout with the naked eye or a camera (Fig. 1). Both heating chambers included a metal heating block and a heated lid. The heating blocks were fabricated by machining aluminum blocks to house 2 and 1.5 mL microcentrifuge tubes in the lysis heating block and 0.1 mL PCR tube strips in the isothermal heating block, respectively. The bottom portion of the heating blocks was machined to attach two ceramic heating elements (24 V, 40 W) and three thermistors for temperature measurements. To ensure thermal stability, heated lids were fabricated and mounted on top of the heating blocks to enclose the sample tubes during the entire assay duration. The lids were fabricated using polycarbonate material and FDM 3D printing. A flexible heating pad (12 V, 40 W) was placed in the lid and covered with a 1-mm thick aluminum plate. A thermistor was also placed with the heater for temperature measurements. The transilluminator included a pair of 2 × 16 LED arrays mounted on the sides to provide illumination from the sides to the LAMP tubes placed at the center. The LEDs were 5 mm in diameter and had 470 nm emission wavelength (C5SMF-BJE-CR0U0451-ND, Digikey, Canada). A non-heated lid was mounted on top of the transilluminator with an optical window covered by an orange acrylic sheet band pass filter. During the end point visualization, the acrylic sheet selectively blocked the light wavelengths lower than 500 nm (including the light emitted by the LED’s). The fluorescence emission from the excited intercalating dye had longer wavelength (*λ*_max_ = 520 nm) and could be transmitted through the acrylic sheet and used for naked-eye readout of fluorometric results. The entire device was controlled using an ESP32 microcontroller (ESP-WROOM-32, Espressif, China). Custom-designed printed circuit boards (PCBs) were used to mount all the required electronic components for controlling the device. An LCD display was used to display current status of the device and available functions. A set of five push buttons was provided for interaction with the user for the White Pearl device operation. A custom-made software was executed on the microcontroller to continuously monitor the temperature data and control the respective heaters. The operation of the heaters (lysis and isothermal modules) was kept sequential and not simultaneously. This allowed to activate them at different intervals during assay workflow and avoid excessive power consumption. The software allowed for changing the lysis and isothermal heating block temperatures during the device operation. Nevertheless, default temperature values of 95°C and 64°C were maintained on the lysis and isothermal heating blocks as mentioned above. The heated lids were maintained at a fixed temperature of 105°C. The temperature in respective heaters was maintained by relay-controlled heating elements and recording temperature feedback from multiple thermistors. The feedback temperature values were measured as the average of multiple readings. A 144 W AC-DC power adapter of 110–220 V input and 24 V/6 A output was used to power the device. Upon turning the heaters on, a warm-up duration of approximately 10 min was required. Since LAMP assays are highly sensitive to fluctuations in incubation temperature, a narrow fluctuation range was specified in the device. This contained the temperature fluctuations in the heaters within the range of ±1°C. The device’s capital cost was approximately US$210.

### LAMP panel for ESBL and carbapenemase gene detection

To screen for the most prevalent ESBL/carbapenemase genes, we designed the URine, ESBL, and CArbapenemase (URECA)-LAMP panel as an 8-tube strip. Each tube contained LAMP chemistry and primers to detect ESBLs and carbapenemase genes in simplex LAMP reactions (Fig. S1). Primers for the detection of ESBL (*bla*_CTX-M-1_-group and *bla*_CTX-M-9_-group) and carbapenemase genes (*bla*_KPC_, *bla*_NDM_ *bla*_OXA-23_, *bla*_OXA-48_-like, and *bla*_VIM_) in *E. coli*, *K. pneumoniae*, *P. aeruginosa,* and *A. baumannii* were adopted from previous publications (25–29). In addition, a newly designed primer assay for the detection of pan-bacterial 16S rRNA as an amplification control was included (Table S1).

The design of new LAMP primers for 16S rRNA was based on a multiple sequence alignment of reference sequences available in NCBI’s Nucleotide databases (30). Alignments were made in Benchling (https://benchling.com) using the MAFFT algorithm with default options (31). The alignments included the 16S rRNA gene sequences of pathogens *E. coli* (NCBI Reference Sequence (NR) NR_112558.1), *K. pneumoniae* (NR_037084.1), *P. aeruginosa* (NR_026078.1), and *A. baumannii* (NR_113237.1), and the sequences of *Lactobacillus iners* (NR_036982.1) and *L. crispatus* (NR_041800.1). These *Lactobacillus* species have been reported as common bacterial flora in urine specimens (32). The alignment was exported from Benchling as “.clustal” files and uploaded to Primer Explorer V4 to obtain newly designed primer sequences (https://primerexplorer.jp/e/). Default Parameter Set and Design Option “Common” were used during primer design. A single-candidate assay that included forward and reverse outer primers (F3 and B3), forward and reverse inner primers (FIP and BIP), and forward and reverse loop primers (LF and LB) was obtained. The single-candidate assay was screened *in silico* using BLAST (30) and tested empirically during fluorometric assays with a CFX-96 instrument (Bio-Rad Laboratories Inc., Hercules, USA).

Individual LAMP reactions for each of the targets in the panel consisted of 12.5 µL of WarmStart LAMP 2 × Mastermix (New England Biolabs Ltd., USA), 0.5 µL of LAMP Fluorescent Dye, 2.5 µL of 10 × primers mix containing 32 µM FIP/BIP, 4 µM F3/B3 and 14 µM LoopF/LoopB, 2.5 µL of 6 µM hydroxynaphthol blue (HNB), 2 µL of nuclease-free water and 5 µL of DNA template, or nuclease-free water for negative controls. URECA-LAMP screening occurred in simplex LAMP reactions and each tube in the 8-tube strip had an individual primer mix for each of the eight targets in URECA-LAMP. All targets in the entire panel were tested with positive control isolates using a CFX real-time instrument.

### Integrating machine-learning algorithm with LAMP for interpretation of LAMP results

This section describes the application of the machine-learning approach to address the subjectivity inherent in visually interpreting LAMP reactions by integrating LAMP with the You Only Look Once (YOLOv8) algorithm, a state-of-the-art multi-object detection model (33). The YOLOv8 algorithm was used to (i) detect individual tubes of LAMP reactions and (ii) classify them into positive or negative reactions within 8-tube strip images of the URECA panel.

#### Data set preparation

Our data set comprised annotated images of LAMP tube samples captured using smartphones. Images from both Android and iOS operating systems were taken with Motorola One 5g Ace and iPhone XR, respectively. Each image was annotated with bounding boxes manually using Computer Vision Annotation Tool (CVAT). Our training data set comprised a diverse range of images representing both positive and negative LAMP results. Classification into positive or negative reactions was objectively determined based on whole-genome sequencing (WGS) results of bacterial isolates, intentionally including examples that may deemed subjective or challenging for visual human interpretation. Classes and image labels were saved in YOLO format.

#### Preprocessing

Prior to model training, the data set underwent preprocessing steps, including normalization to standardize pixel intensities. This preprocessing step is important for optimizing model convergence, reducing sensitivity to lighting variations, and enhancing numerical stability in the YOLOv8 model.

#### Model architecture and training

The YOLOv8 algorithm, due to its efficient processing of the entire input image in a single pass through a convolutional neural network (CNN), was selected for its lightweight network architecture and superior detection accuracy. The model was trained on a computer equipped with an NVIDIA RTX 3060 12 GB GPU, Intel i7-10700F CPU operating at 2.90 GHz, 64 GB of RAM, and Windows 10 64-bit operating system. Python version 3.11 and the PyTorch deep learning framework were used for script execution. The training process utilized images resized to 640 × 640, a batch size of 8, a learning rate of 0.01, and 80 epochs. The data set was randomly divided into three sets: training (85%) and validation (15%), consisting of 3,018, and 566 images, respectively. To enhance the model’s generalizability and robustness, data augmentation techniques were applied to the training set, expanding it to 4638 images.

The training process used images of full 8-tube strips; since the problem is formulated as an object detection task (i.e., localization of tubes and classification), the algorithm is trained on tubes annotated with bounding boxes and labels indicating its classification. Once the model is trained, the overall number of tubes in any single image is less critical, as YOLO is designed to autonomously detect and recognize any number of tubes present in a LAMP image.

**Fig 2:**
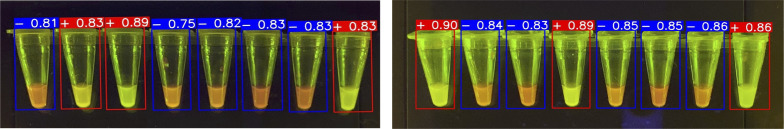
Detection results of YOLOv8 applied on LAMP images. Two example images from a commercial transilluminator demonstrate the capacity of the algorithm to detect and classify positive (fluorescent green) and negative (dull orange) results of individual wells in 8-tube LAMP strips. The positive samples are marked with red bounding boxes, while the negative samples are highlighted with blue bounding boxes. Each detected sample in the images is assigned a confidence score/probability that reflects the model’s certainty that a given detection corresponds to a real object and its corresponding class. A confidence threshold above 0.5 was utilized in the algorithm to determine the validity of the detections and their classifications.

The trained algorithm classified individual tubes in 8-tube strips as exemplified in Fig. 2.

#### Evaluation metric

As an object detection problem, YOLOv8 performs both localization and classification simultaneously, and therefore, a confusion matrix was used for its performance evaluation. The model’s performance was evaluated using precision, recall, and F-1 score metrics from the confusion matrix. Precision measures the proportion of correctly detected targets among those detected by the model. Recall is defined as the ratio of correctly identified positive targets to the total number of actual positive targets. The F1-score is a metric that combines both precision and recall, calculated as the harmonic mean of these two values (34). These values are particularly relevant for assessing the balance between sensitivity and specificity for reliable screening results.

Additionally, we evaluated the accuracy metric, which provides a comprehensive measure of the overall correctness of the model’s predictions. Accuracy is calculated as the ratio of correctly classified predictions to the total number of object predictions.

#### Implementation as smartphone app

To facilitate deployment and accessibility, the evaluated YOLOv8 model was integrated into a custom smartphone application developed using Kotlin and Java programming languages within the Android Studio environment. This leverages smartphone capabilities for on-site LAMP analysis through the camera. The user-friendly interface allows capturing images of URECA panels and displaying the model’s predictions of AMR results, including genes concomitantly present in individual samples. Photographic images were obtained with the White Pearl instrument transilluminator for analysis using the YOLOv8 model.

### Validation of NPT platform

#### Clinical isolates

A sample set of 53 unique clinical isolates obtained from Diagnostic and Scientific Centre (Calgary, Alberta, Canada) and the SMART and INFORM bacterial collections (35, 36) (Table S2) was used during validation. The isolates were subjected to whole-genome sequencing (WGS) as part of previous publications (37–42). At least 10 isolates carrying the ESBL and carbapenemase genes in the URECA panel were used: *bla*_CTX-M-1_-group (*n* = 13), *bla*_CTX-M-9_-group (*n* = 11), *bla*_OXA-48_-like (*n* = 14), *bla*_NDM_ (*n* = 11), *bla*_VIM_ (*n* = 11), *bla*_OXA-23_ (*n* = 10), and *bla*_KPC_ (*n* = 10). Among the 53 isolates, 20 carried a combination of multiple beta-lactamases: *bla*_CTX-M-1_-group, *bla*_CTX-M-9_-group, *bla*_NDM_ (*n* = 4); *bla*_CTX-M-1_-group, *bla*_OXA-48_-like (*n* = 4); *bla*_CTX-M-9_-group, *bla*_OXA-48_-like (*n* = 3); *bla*_CTX-M-1_-group, *bla*_NDM_ (*n* = 2); *bla*_OXA-48_-like, *bla*_VIM_ (*n* = 2); *bla*_CTX-M-1_-group, *bla*_OXA-48_-like, *bla*_NDM_ (*n* = 1); *bla*_NDM_, *bla*_KPC_ (*n* = 1); *bla*_OXA-48_-like, *bla*_NDM_, *bla*_VIM_ (*n* = 1); *bla*_NDM_, *bla*_OXA-23_ (*n* = 1); *bla*_CTX-M-1_-group, *bla*_CTX-M-9_-group, *bla*_VIM_ (*n* = 1) (Table S2).

**Fig 3:**
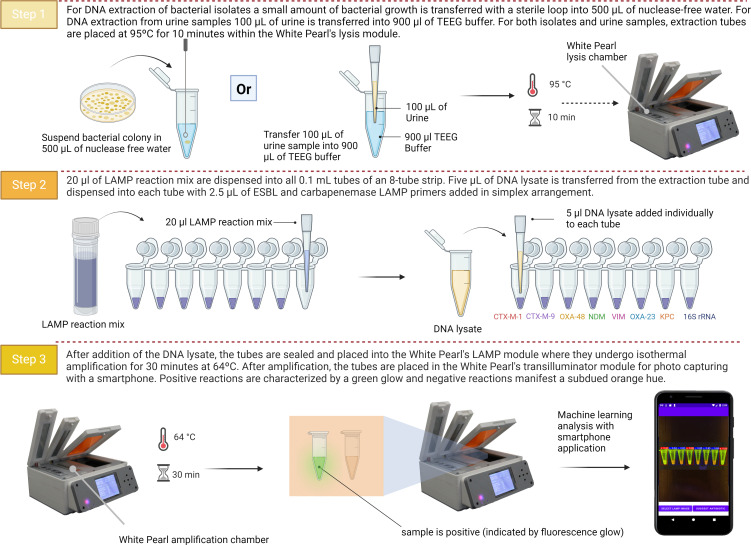
URECA-LAMP platform workflow using isolates and urine samples. Step 1, sample preparation and DNA extraction. Step 2, preparation of LAMP reactions and addition of DNA lysate. Step 3, LAMP incubation and photo capture with the smartphone app. Created in BioRender. Pillai, D. (2024) BioRender.com/w91a325.

A single sweep of bacterial colonies was taken using a loop and suspended in a 1.5 mL microcentrifuge tube with 500 µL of sterile nuclease-free water. Bacterial cells were thoroughly mixed by twirling a loop inside the tube. The 500 µL bacterial suspension was then lysed at 95°C for 10 min in the heating block of the White Pearl. Lysates were allowed to cool down for 10 min before using as a template in LAMP reactions. As lysates cooled down, LAMP reactions were prepared as explained above and 10× primer stocks for each of the targets were dispensed individually in each of the wells of the LAMP strip. Five microliters of lysate was dispensed individually into each of the LAMP reactions in the URECA-LAMP strip, mixed by gently pipetting up and down, and then incubated at 64°C for 30 min in the White Pearl’s isothermal heating block. After 30 min, the URECA-LAMP strips were taken out of the isothermal heating block and placed inside the transilluminator of the White Pearl device under the blue LED light. LAMP results were visually assessed through the orange filter on the lid of the transilluminator, and a photo was captured for interpretation with the smartphone application. The URECA-LAMP platform, consisting of the White Pearl device, URECA panel, and smartphone application, was used to screen ESBL/carbapenemase-carrying isolates and contrived urine samples. All sample-to-result steps were performed on the White Pearl device, and interpretation of LAMP results was done with the YOLOv8 algorithm. The platform’s workflow is shown in Fig. 3.

Initial assessment of the results was made with naked eye fluorometric interpretation. Discrepant results between inital testing with the White Pearl and WGS were resolved by repeating the testing with the White Pearl. Initial concordant results and the results of repeat tests were analayzed using the YOLOv8 algorithm. In order to determine positive percent and negative percent agreements (PPA and NPA), WGS results were used as the gold standard.

#### Contrived urine specimens

A sweep of bacterial colonies was suspended in nuclease-free water and standardized to an Optical Density of 1 at 600 nm (OD600 = 1). From the bacterial suspension with OD600 = 1, 100 µL was aded to 100 µL of uninfected normal urine, and 900 µL of TEEG buffer (10 mM Tris, 1 mM EDTA, 1 mM EGTA). This mixture was vortexed and then lysed at 95°C for 10 min in the White Pearl’s heat lysis block, followed by the URECA-LAMP testing steps shown in Fig. 3.

#### Limit of detection studies with contrived urine specimens

Contrived urine samples were prepared as mentioned above with exception that 10μL of bacterial suspension was used. Seven 10-fold serial dilutions (10^−1^ to 10^−7^) of bacteria were prepared in nuclease-free water. In order to quantify the bacterial count, 100 µL of the 10^−4^ to 10^−7^ dilutions was plated in triplicate in Luria Berthani (LB) agar to determine the number of colony-forming units (CFUs/mL). The contrived samples were vortexed and then lysed at 95°C for 10 min in the White Pearl’s lysis block. Next, 5 µL of lysates was used as a template and directly distributed into LAMP reactions as shown in Fig. 3. In an effort to improve the LOD values of *bla*_CTX-9_ and *bla*_VIM_, an additional centrifugation step of the lysate at 14,000 RCF for 2 min was introduced. A total of four technical replicates per dilution was tested. The LOD of individual targets was defined as the highest dilution (lowest concentration) in which all replicates (4/4) showed a positive result.

## RESULTS

### LAMP panel for ESBL and carbapenemase gene detection

Each tube contained LAMP chemistry and primers to detect the most relevant ESBLs and carbapenemase genes. A newly designed primer assay for the detection of pan-bacterial 16S rRNA as an amplification control was included in the panel (Table S1). Time-to-positivity was under 20 min for every target using a CFX real-time instrument.

### Training and validation of machine-learning algorithm

**Fig 4:**
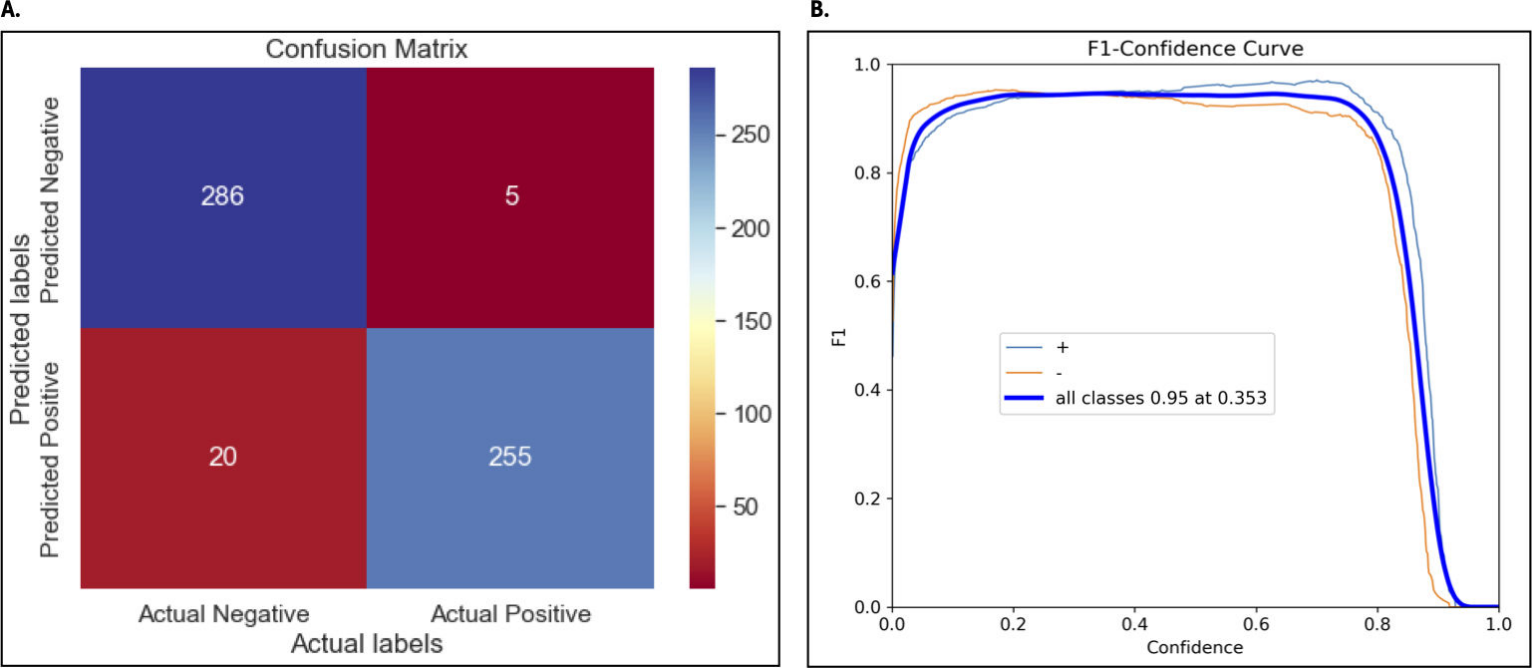
Performance metrics obtained from the trained algorithm. (**A**) Confusion matrix shows the number of correctly classified and misclassified samples for both positive and negative classes. (**B**) F1-score for all classes across different confidence thresholds on the validation data set with an F1-score of 95% for all classes at a confidence threshold of 0.353.

Once trained, the YOLOv8 algorithm was used to classify LAMP reactions based on the validation data set. The overall accuracy of 95.5% was achieved to detect both positive and negative samples. Additionally, 98.0% precision and 92.7% recall for positive cases and 93.4% precision and 98.2% recall for negative cases was observed. The F1-score of 95.3% for the positive class and 95.8% for the negative class was obtained (Fig. 4).

After evaluating the performance of the model, the model was integrated into a custom smartphone application developed using Kotlin and Java programming languages within the Android Studio environment. The user interface comprises four screens as shown in Fig. S2.

The architecture of the smartphone application is designed to be modular, enabling seamless updates to the core YOLOv8 model without necessitating a complete system overhaul. This design facilitates the integration of advancements in the YOLO algorithm and ensures the application remains current with the latest developments in object detection technology. To adapt to new YOLO versions, periodic training of the model with new LAMP test images can be made using Transfer Learning techniques, enhancing the model’s detection capabilities in real-world conditions.

### Validation of NPT platform using clinical isolates and urine specimens

Validation was performed using a sample set of 53 unique clinical isolates as well as SMART and INFORM bacterial collections (35, 36) (Table S2). At least 10 isolates carrying the ESBL and carbapenemase genes in the URECA panel, *bla*_CTX-M-1_-group, *bla*_CTX-M-9_-group, *bla*_OXA-48_-like, *bla*_NDM_, *bla*_VIM_, *bla*_OXA-23_ and *bla*_KPC_, were used. Compared to WGS, URECA-LAMP achieved a PPA of 100% for all targets. NPA was 100% for all targets except for *bla*_CTX-M-9_-group with 95.2% (Table 1; Table S3).

**TABLE 1:** Summary of positive percent agreement and negative percent agreement for URECA gene targets from bacterial isolates^*a*^

WGS-detected gene	Positive percent agreement (95% CI)	Negative percent agreement (95% CI)
*bla*_CTX-M-1_-group (*n* = 13)	100 (77.2–100)	100 (91.2–100)
*bla*_CTX-M-9_-group (*n* = 11)	100 (74.1–100)	95.2 (84.2–98.7)
*bla*_OXA-48_-like (*n* = 14)	100 (78.5–100)	100 (91.0–100)
*bla*_NDM_ (*n* = 11)	100 (74.1–100)	100 (91.6–100)
*bla*_VIM_ (*n* = 11)	100 (74.1–100)	100 (91.6–100)
*bla*_OXA-23_ (*n* = 10)	100 (72.3–100)	100 (91.8–100)
*bla*_KPC_ (*n* = 10)	100 (72.3–100)	100 (72.3–100)

^*a*^ PPA and NPA were calculated using true positives (TP), true negatives (TN), false positives (FP), and false negatives (FN) values. PPA = TP/TP + FN and PNA = TN/TN + FP. WGS was treated as the gold standard. Results from contrived specimens can be found in the supplemental material.

To evaluate the suitability of the platform for screening human specimens, contrived urine specimens were used (*n* = 3 isolates per ESBL and carbapenemase target). Contrived samples were evaluated following the same workflow in Fig. 3. A total of 21 contrived samples was evaluated, and all were correctly detected (Table S4). Additionally, 21 ‘normal’ uninfected urine samples were tested with the platform. One ‘normal’ urine sample was positive for *bla*_CTX-M-1_-group gene.

Following the workflow for urine samples in Fig. 3, the LOD was confirmed at 10^4^, 10^7^, 10^4^, 10^4^, 10^7^, 10^3^, and 10^6^ CFU/µL for CTX-M-1, CTX-M-9, OXA-48, NDM, VIM, OXA-23, and KPC, respectively. The introduction of a centrifugation step for CTX-M-9 and VIM resulted in lower LOD values of 10^5^ for these two targets (Table S5).

## DISCUSSION

We developed and validated a platform for the rapid, robust, and low-cost detection of ESBL and carbapenemase genes in critical priority pathogens. The different tools, which include the White Pearl device, the URECA panel, and the smartphone application for interpretation of LAMP results, were combined to provide PPA and NPA values of 100% during validation with clinical isolates. The only target below this value was the *bla*_CTX-M-9_-group with NPA of 95.2%. Looking into the interpretation images of the YOLO algorithm, the reduced NPA values were likely a misclassification of the algorithm in 2 out of 11 *bla*_CTX-M-9_-group isolates tested. Machine-learning algorithms improve their classifying capacity as more data are used for training and testing. We anticipate that as the algorithm continues to be used and more LAMP images and interpretation results are generated, the NPA value for this target will increase. Besides the screening of pure bacterial cultures, the evaluation of contrived urine samples demonstrated the suitability of the platform to test this type of human specimen. All contrived specimens yielded positive results for the targets they were tested. Overall, the PPA, NPA, and the metrics used to evaluate accuracy, precision, and recall of the algorithm, indicated the suitability of the platform to detect ESBL and carbapenemase genes when screening bacterial isolates and urine specimens.

Several reports of LAMP-based assays using near patient or POC devices and smartphone applications are available (43). However, none of them have been validated for the detection of ESBLs or carbapenemase genes. It was, therefore, not possible to compare these platforms with our platform. Our assay achieved comparable performance to previous LAMP assays for the detection of ESBLs and carbapenemases in Gram-negative bacteria (26–29). In these reports, values of 100% agreement were achieved during validation with DNA extracts from pure bacterial cultures. Another assay comparable to ours is the CE-IVD LAMP Eazyplex SuperBug kits (Amplex-Diagnostics GmbH, Germany). This commercial assay is based on real-time detection of LAMP products and designed to detect specific ESBL and carbapenemase genes using bacterial isolates or human specimens as samples. When assessed on clinical isolates of Enterobacterales, sensitivity and specificity values of Eazyplex SuperBug were greater than 95% (44, 45). Although highly sensitive and specific, this assay requires the use of specialized and costly devices with real-time capabilities. In contrast, the advantages of our platform are (i) its reduced cost with per-device capital cost of approximately US$210 and US$16.7 per URECA test (8-tube strip) which can be further reduced with in house enzyme production and (ii) the visual interpretation of fluorometric results with the naked eye or the smartphone application we developed. In addition, the targets in the LAMP panel in this study are customizable and can be adjusted to other AMR markers or microbial species.

In general, one of the limitations of LAMP is the subjectivity of naked eye interpretation of results for non-laboratorians or unskilled personnel. Given the high amounts of amplicons produced, LAMP readouts can lead to false positives (23, 46). Previous studies reported the use of HNB as a pH-based colorimetric indicator to increase discrimination between positive and negative colorimetric readouts (27). In our case, and as previously reported by our group, HNB was used to improve visual fluorometric readout by reducing the signal emitted by free fluorescent dye in negative reactions (24). Furthermore, a major novelty of our study was the development of the machine-learning smartphone app for the interpretation of URECA-LAMP results. The development of a new smartphone app has been recommended to increase sensitivity and specificity of LAMP assays (24, 29). Owing to its multi-object detection and classification capability, the YOLOv8 algorithm was efficient in accurately categorizing multiple tube images within a photograph in a single pass. The reliability offered by interpretation with the smartphone application increases the feasibility of bringing URECA-LAMP from near-the-patient to the point-of-care by reducing the need for highly skilled personnel to interpret the results.

One hallmark of the White Pearl device is its simple DNA extraction methodology where no centrifugation or DNA purification is required. DNA extraction methodologies have been shown to influence the analytical sensitivity of carbapenemase detection assays (27). In our case, *bla*_CTX-M-9_-group and *bla*_VIM_ were the targets with the lowest analytical sensitivity when testing contrived urine samples (10^5^ CFU/mL, with centrifugation), while *bla*_OXA-23_ had the highest (10^3^ CFU/mL). In general, higher analytical sensitivity can be achieved through different strategies, including alternative DNA extraction methodologies, dilution of inhibitory substances, or use of different primer sequences (27). Future optimization work in our laboratory include the use of point-of-care extraction methodologies to enhance the LOD values of all targets in our assay. Nevertheless, the observed LOD values in our assay did not diminish the PPA values for any of the targets in the URECA panel. Thus, we can conclude the analytical sensitivity of URECA-LAMP assay is at a sufficent level for gene detection in bacterial isolates and contrived urine samples in the clinical setting.

One of the limitations of URECA-LAMP is its low-throughput format since only one sample can be run at a time. Nevertheless, the low cost and portability of the White Pearl device would enable users to operate more than one device in the laboratory. It is also important to consider that portable assays such as URECA-LAMP are ultimately intended for POC use, a scenario where high throughput is not a requirement.

Future work on URECA-LAMP include its adaptation to test different types of human specimens. We anticipate the URECA panel can be used to screen for ESBL and carbapenemase genes in respiratory tract infections, blood stream infections, and fecal colonization. Ongoing work in our laboratory include the development and validation of spin-free DNA extraction that could be adapted to work with this specimen types as well as the low-cost lyophilization of LAMP reagents. These steps are required to achieve POC and specimen-to-result suitability. To confirm the suitability of the platform for work in the clinical setting, future work should also include the validation of the LAMP result interpretation module to guide clinicians on their choices based on URECA-LAMP diagnosis (Fig. S2).

In conclusion, the URECA-LAMP platform is suitable for the detection of ESBLs and carbapenemases in clinical isolates and urine specimens. The platform leverages the advantages of LAMP for the detection of ESBLs and carbapenemases using the White Pearl device. In addition, the newly developed smartphone application helps surpass the limitations of visual interpretation of LAMP results by non-laboratorians. The reduced cost and portability of the URECA-LAMP make it attractive for implementation in a variety of scenarios in high and low middle-income countries to support antimicrobial stewardship.

## Data Availability

The code and data used in this study are available upon request.

## SUPPLEMENTAL MATERIAL

Supplemental material jcm.00869-24-s0001.docx Figures S1 and S2; Tables S1 to S5.

ASM does not own the copyrights to Supplemental Material that may be linked to, or accessed through, an article. The authors have granted ASM a non-exclusive, world-wide license to publish the Supplemental Material files. Please contact the corresponding author directly for reuse.
