# Supplementary material for: A novel machine-learning aided platform for rapid detection of urine ESBLs and carbapenemases: URECA-LAMP
Source: J Clin Microbiol. 2024 Oct 24;62(11):e00869-24. doi: 10.1128/jcm.00869-24 (PMC11559160; doi:10.1128/jcm.00869-24)
Supplement: Supplemental material — Figures S1 and S2; Tables S1 to S5. [file jcm.00869-24-s0001.docx]

**Supplementary Figure 1 - URECA panel strip.** 8-tube PCR strips contained 25 μL LAMP reactions in each 0.1 mL well. The different targets were screened in simplex LAMP reactions. Fluorescent green indicates a positive result while dull orange indicates a negative result. In this exemplifying image, the test is positive for *bla*_CTX-M-1-group,_ *bla*_OXA-48_-like and 16S rRNA amplification control.

**
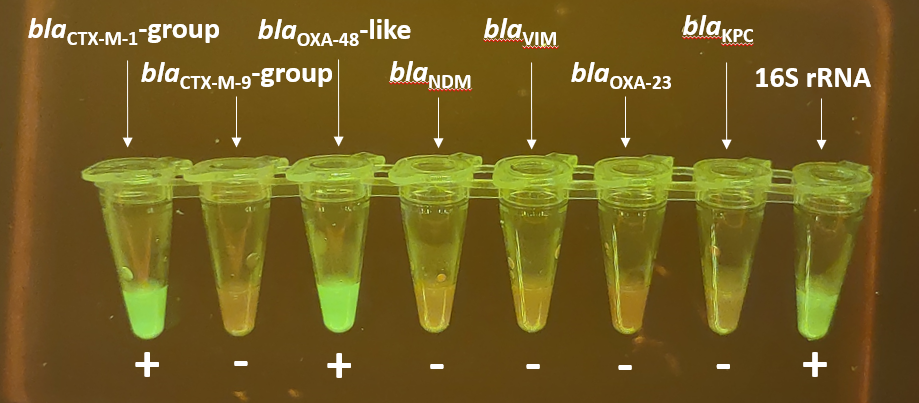
**

**Supplementary Figure 2 - Smartphone application for LAMP reaction analysis.** (A) User select the panel and sample type they want to analyze (B) User choose a LAMP image from the phone’s storage (C) the trained model automatically detects and classified the tube samples within the image and (D) upon clicking “Suggest Antibiotic”, the application displays the recommended guidelines based on the LAMP results.

**Supplementary Table 1 – ESBL and carbapenemase targets and primer sequences in URECA-LAMP.**

| **URECA gene target** | **Primer** | **Sequence** | **Reference** |
| --- | --- | --- | --- |
| *bla*_CTX-M-1-group_ | F3 | AACTCTGCGGAATCTGAC | Anjum et al, 2013 |
|  | B3 | TTTCTGCCTTAGGTTGAGG |  |
|  | FIP | CCCACAACCCAGGAAGCAAAAAGCTGGTGACATGGATGAA |  |
|  | BIP | GTGGCTATGGCACCACCAAAAAGTAAGTGACCAGAATCAGC |  |
|  | LF | AGTCCAGCCTGAATGCTCG |  |
|  | LB | ACGATATCGCGGTGATCTGG |  |
|  |  |  |  |
| *bla*_CTX-M-9-group_ | F3 | TGCGCTGGGCGAAACC | Rivoarilala et al, 2018 |
|  | B3 | GGCTCTCTGCGTTCTGTTG |  |
|  | FIP | GGTAAGCCGGCCCGAATGCTTTTTGTTGGTGACGTGGCTCAA |  |
|  | BIP | GGCACCACCAATGATATTGCGGCGGCTGGGTAAAATAGGTCA |  |
|  | LF | CGCCGGTCGTATTGCCT |  |
|  | LB | TGATCTGGCCGCAGGGT |  |
|  |  |  |  |
| *bla*_OXA-48-like_ | F3 | ATCACAGGGCGTAGTTGT | Poirier et al, 2022 |
|  | B3 | CGTCTGTCCATCCCACTT |  |
|  | FIP | TGCTTGGTTCGCCCGTTTAACTCTGGAATGAGAATAAGCAG |  |
|  | BIP | TACCCGCATCTACCTTTAAAATTCCAAGACTTGGTGTTCATCCT |  |
|  | LB | GCTTGATCGCCCTCGATTTG |  |
|  |  |  |  |
| *bla*_NDM_ | F3 | CATTAGCCGCTGCATTGATG | Srisrattakarn et al, 2017 |
|  | B3 | CCGCCATCCCTGACGATC |  |
|  | FIP | ATCGCCAAACCGTTGGTCGCCCGGTGAAATCCGCCCG |  |
|  | BIP | TGGTTTTCCGCCAGCTCGCAGCGACTGCCCCGAAAC |  |
|  | LF | TCCATTTGCTGGCCAATCG |  |
|  | LB | ACCGAATGTCTGGCAGCACA |  |
|  |  |  |  |
| *bla*_VIM_ | F3 | GTTTGGTCGCATATCGCAACG | Srisrattakarn et al, 2017 |
|  | B3 | CCAGCCGCCCGAAGGACATC |  |
|  | FIP | CGCACCCCACGCTGTATCAATCGTCTACCCGTCCAATGGTCTC |  |
|  | BIP | GCGGCACTTCTCGCGGAGATTGACCGCCGACGCGGTCGTCATG |  |
|  | LF | CAACTCATCACCATCACGGAC |  |
|  | LB | GTAACGCGTGCAGTCTCCAC |  |
|  |  |  |  |
| *bla*_OXA-23_ | F3 | GAAGCCATGAAGCTTTCTG | Yamamoto et al, 2015 |
|  | B3 | GTATGTGCTAATTGGGAAACA |  |
|  | FIP | ACCGAAACCAATACGTTTTACTTCTCAGTCCCAGTCTATCAGGA |  |
|  | BIP | CTGAAATTGGACAGCAGGTTGACTCTACCTCTTGAATAGGCG |  |
|  | LF | TTTTGCATGAGATCAAGACCGA |  |
|  | LB | CTGGTTGGTAGGACCATTAAAGGTT |  |
|  |  |  |  |
| *bla*_KPC_ | F3 | TGGACACACCCATCCGT | Feng et al, 2021 |
|  | B3 | GGAACGTGGTATCGCCG |  |
|  | FIP | ACCGTCATGCCTGTTGTCAGATCGGCAAAAATGCGCTGGT |  |
|  | BIP | AATACAGTGATAACGCCGCCGCCGCATGAAGGCCGTCAG |  |
|  | LF | GAGATGGGTGACCACGGA |  |
|  | LB | CAATTTGTTGCTGAAGGAGTTGGGC |  |
|  |  |  |  |
| 16S rRNA | F3 | GTGGGGAGCAAACAGGATT | *Present study* |
|  | B3 | TCTTCGCGTTGCTTCGAATT |  |
|  | FIP | AGGCGGTCGACTTATCGCGTTAGTCCACGCCGTAAACGATG |  |
|  | BIP | GGAGTACGGCCGCAAGGTTAACATGCTCCACCGCTTGTG |  |
|  | LF | CAAGGATCCCAACGGCTAGT |  |
|  | LB | AACTCAAATGAATTGACGGGGGC |  |

**Supplementary Table 2 – List of isolates used during URECA-LAMP validation.**

| **Strain name** | **Species** | **Previously WGS detected ESBL/carbapenemase genes in URECA** | **URECA-LAMP results** | **Publication reference** | **WGS accession number** | **Country of origin** | **Year of collection** | **Type of infection** |
| --- | --- | --- | --- | --- | --- | --- | --- | --- |
| 15.5635.2 | *A. baumannii* | OXA-23 | OXA-23 | unpublished | not submitted | Canada | 2015 | respiratory infection |
| 17.1668 | *A. baumannii* | OXA-23 | OXA-23 | unpublished | not submitted | Mexico-returning traveller | 2017 | bloodstream infection |
| 17.9669 | *A. baumannii* | OXA-23 | OXA-23 | unpublished | not submitted | Canada | 2017 | wound |
| AB 01 | *A. baumannii* | OXA-23 | OXA-23 | PMID: 23612195 | not submitted | Canada | 2012 | wound |
| AB 02 | *A. baumannii* | OXA-23 | OXA-23 | PMID: 23612195 | not submitted | Canada | 2012 | wound |
| AB 03 | *A. baumannii* | OXA-23 | OXA-23 | PMID: 23612195 | not submitted | Canada | 2012 | wound |
| AB 04 | *A. baumannii* | OXA-23 | OXA-23 | PMID: 23612195 | not submitted | Canada | 2012 | wound |
| AB QC | *A. baumannii* | OXA-23 | OXA-23 | unpublished | not submitted | France | 2011 | unknown |
| 20-8958 | *A. baumannii* | OXA-23 | OXA-23 | unpublished | not submitted | Canada | 2020 | urinary infection |
| 20-01 | *A. baumannii* | OXA-23, NDM | OXA-23, NDM | unpublished | not submitted | Canada | 2020 | urinary infection |
| AZ 30 | *K. pneumoniae* | VIM-1 | VIM | unpublished | not submitted | Greece | 2012 | urinary infection |
| AZ 58 | *K. pneumoniae* | VIM-1 | VIM | PMID: 28520983 | DRR065653 | Greece | 2012 | Furuncle |
| AZ 241 | *K. pneumoniae* | CTX-M-15 (CTX-M-1) | CTX-M-1 | unpublished | not submitted | Romania | 2013 | respiratory infection |
| AZ 564 | *Enterobacter* | VIM-23 | VIM | PMID: 29774858 | SAMN07501510 | Mexico | 2012 | skin ulcer |
| AZ 593 | *K. pneumoniae* | OXA-48 | OXA-48 | unpublished | not submitted | Turkey | 2012 | urinary infection |
| AZ 594 | *Enterobacter* | VIM-31, OXA-48 | VIM, OXA-48 | PMID: 29774858 | SAMN07501515 | Turkey | 2012 | urinary infection |
| AZ 661 | *Enterobacter* | VIM-4, OXA-48 | VIM, OXA-48 | PMID: 29774858 | SAMN07501529 | Kuwait | 2013 | wound |
| AZ 668 | *K. pneumoniae* | OXA-48 | OXA-48 | unpublished | not submitted | Spain | 2013 | skin ulcer |
| AZ 793 | *K. pneumoniae* | OXA-181 (OXA-48), CTX-M-15 (CTX-M-1) | OXA-48, CTX-M-1 | unpublished | not submitted | Kenya | 2014 | respiratory infection |
| AZ 800 | *Enterobacter* | NDM-7 | NDM | PMID: 29774858 | SAMN07501549 | Philippines | 2014 | wound |
| AZ 860 | *K. pneumoniae* | CTX-M-1, NDM, OXA-48 | CTX-M-1,NDM,OXA-48 | unpublished | not submitted | Romania | 2014 | abscess |
| AZ 886 | *Enterobacter* | VIM-23 | VIM | PMID: 29774858 | SAMN07501559 | Mexico | 2014 | respiratory infection |
| AZ 890 | *Enterobacter* | VIM-1 | VIM | PMID: 29774858 | SAMN07501561 | Greece | 2014 | pancreatic infection |
| ESBL 10.573 | *E. coli* | CTX-M-24 (CTX-M-9) | CTX-M-9 | PMID: 22162555 | not submitted | Canada | 2010 | bloodstream infection |
| EC 16.149 | *E. coli* | CTX-M-27 (CTX-M-9) | CTX-M-9 | PMID: 32120035 | not submitted | Canada | 2016 | bloodstream infection |
| EC 16.088 | *E. coli* | CTX-M-14 (CTX-M-9) | CTX-M-9 | PMID: 32120035 | not submitted | Canada | 2016 | bloodstream infection |
| KP SA01 | *K. pneumoniae* | VIM-1 | VIM | PMID: 28520983 | DRR065595 | South Africa | 2010 | Bodily fluids: Abscess/pus |
| SM 02 | *K. pneumoniae* | VIM-27 | VIM | PMID: 28520983 | DRR065656 | Greece | 2008 | intra-abdominal infection |
| SM 110 | *K. pneumoniae* | OXA-48, CTX-M-15 (CTX-M-1) | OXA-48, CTX-M-1 | unpublished | not submitted | Turkey | 2009 | intra-abdominal infection |
| SM 112 | *K. pneumoniae* | OXA-48, CTX-M-15 (CTX-M-1) | OXA-48, CTX-M-1 | unpublished | not submitted | USA | 2009 | intra-abdominal infection |
| SM 115 | *K. pneumoniae* | OXA-181 (OXA-48), CTX-M-15 (CTX-M-1) | OXA-48, CTX-M-1 | unpublished | not submitted | India | 2009 | intra-abdominal infection |
| SM 372 | *K. pneumoniae* | VIM-29, CTX-M-15 (CTX-M-1), CTX-M-14 (CTX-M-9) | VIM, CTX-M-1, CTX-M-9 | PMID: 28520983 | DRR065611 | Saudi Arabia | 2011 | intra-abdominal infection |
| SM 1060 | *Enterobacter* | OXA-48, CTX-M-14 (CTX-M-9) | OXA-48, CTX-M-9 | PMID: 29774858 | SAMN04252948 | Saudi Arabia | 2013 | unknown |
| SM 1109 | *Enterobacter* | OXA-48 | OXA-48 | PMID: 29774858 | SAMN04252954 | Morocco | 2013 | unknown |
| SM 1158 | *K. pneumoniae* | NDM-1, VIM-1, OXA-244 (OXA-48) | NDM, VIM, OXA-48 | unpublished | not submitted | Turkey | 2014 | urinary infection |
| SM 2100 | *E. coli* | NDM-1, CTX-M-15 (CTX-M-1) | NDM, CTX-M-1 | PMID: 35451367 | not submitted | Spain | 2015 | urinary infection |
| SM 2300 | *E. coli* | NDM-6, CTX-M-15 (CTX-M-1), CTX-M-27 (CTX-M-9) | NDM, CTX-M-1, CTX-M-9 | PMID: 35451367 | not submitted | Guatemala | 2016 | intra-abdominal infection |
| SM 2793 | *E. coli* | CTX-M-14 (CTX-M-9), OXA-244 (OXA-48) | OXA-48, CTX-M-9 | PMID: 35451367 | not submitted | Egypt | 2016 | respiratory infection |
| SM 2825 | *E. coli* | NDM-1, CTX-M-15 (CTX-M-1) | NDM, CTX-M-1 | PMID: 35451367 | not submitted | Egypt | 2016 | intra-abdominal infection |
| SM 3080 | *E. coli* | NDM-4, CTX-M-14 (CTX-M-9), CTX-M-15 (CTX-M-1) | NDM, CTX-M-9, CTX-M-1 | PMID: 35451367 | not submitted | Vietnam | 2016 | intra-abdominal infection |
| SM 3100 | *E. coli* | NDM-5, CTX-M-14 (CTX-M-9), CTX-M-55 (CTX-M-1) | NDM, CTX-M-9, CTX-M-1 | PMID: 35451367 | not submitted | Vietnam | 2016 | respiratory infection |
| SM 3102 | *E. coli* | NDM-5, CTX-M-14 (CTX-M-9), CTX-M-55 (CTX-M-1) | NDM, CTX-M-9, CTX-M-1 | PMID: 35451367 | not submitted | Vietnam | 2016 | respiratory infection |
| SM 3168 | *E. coli* | OXA-48, CTX-M-24 (CTX-M-9) | OXA-48, CTX-M-9 | PMID: 35451367 | not submitted | Israel | 2016 | respiratory infection |
| AZ 190 | *K. pneumoniae* | KPC-3 | KPC | unpublished | not submitted | Italy | 2013 | INT: wound |
| AZ 197 | *K. pneumoniae* | KPC-2 | KPC | unpublished | not submitted | Greece | 2013 | INT: abscess |
| SM 901 | *Enterobacter* | KPC-4 | KPC | PMID: 29774858 | SRR2960137 | Puerto Rico | 2013 | intra-abdominal infection |
| SM 935 | *Enterobacter* | KPC-5 | KPC | PMID: 29774858 | SRR2960139 | Puerto Rico | 2013 | urinary infection |
| SM 454 | *Enterobacter* | KPC-3 | KPC | PMID: 29774858 | SRR2960079 | USA | 2011 | intra-abdominal infection |
| SM 3216 | *E. coli* | KPC-2 | KPC | PMID: 35451367 | SAMN23168889 | Colombia | 2016 | urinary infection |
| SM 1450 | *E. coli* | KPC-3 | KPC | PMID: 35451367 | SAMN23168763 | USA | 2016 | urinary infection |
| SM 3120 | *E. coli* | KPC-2 | KPC | PMID: 35451367 | SAMN23168880 | Colombia | 2016 | urinary infection |
| SM 1351 | *Enterobacter* | KPC-2, NDM-1 | KPC, NDM | PMID: 29774858 | SRR3110109 | Colombia | 2014 | urinary infection |
| AZ 76 | *K. pneumoniae* | KPC-2 | KPC | unpublished | not submitted | Argentina | 2012 | INT: wound |

**Supplementary Table 3 - Contingency tables for individual URECA targets - After repeat testing and YOLOv8 interpretation.**

|  | *bla*_CTX-M-1_-group (n= 13) | URECA-LAMP | |  |  |  |
| --- | --- | --- | --- | --- | --- | --- |
|  | WGS | Positive | Negative | Total |  |  |
|  | positive | 13 | 0 | 13 | 100.00 | PPA - Positive Percent Agreement |
|  | Negative | 0 | 40 | 40 | 100.00 | NPA - Negative Percent Agreement |
|  | Total | 13 | 40 |  |  |  |
|  |  |  |  |  |  |  |
|  | *bla*_CTX-M-9_-group (n= 11) |  | |  |  |  |
|  | WGS | Positive | Negative | Total |  |  |
|  | positive | 11 | 0 | 11 | 100.00 | PPA |
|  | Negative | 2 | 40 | 42 | 95.24 | NPA |
|  | Total | 13 | 40 |  |  |  |
|  |  |  |  |  |  |  |
|  | *bla*_OXA-48_-like (n= 14) | URECA-LAMP | |  |  |  |
|  | WGS | Positive | Negative | Total |  |  |
|  | positive | 14 | 0 | 14 | 100.00 | PPA |
|  | Negative | 0 | 39 | 39 | 100.00 | NPA |
|  | Total | 14 | 39 |  |  |  |
|  |  |  |  |  |  |  |
|  | *bla*_NDM_ (n= 11) | URECA-LAMP | |  |  |  |
|  | WGS | Positive | Negative | Total |  |  |
|  | positive | 11 | 0 | 11 | 100.00 | PPA |
|  | Negative | 0 | 42 | 42 | 100.00 | NPA |
|  | Total | 11 | 42 |  |  |  |
|  |  |  |  |  |  |  |
|  | *bla*_VIM_ (n= 11) | URECA-LAMP | |  |  |  |
|  | WGS | Positive | Negative | Total |  |  |
|  | positive | 11 | 0 | 11 | 100.00 | PPA |
|  | Negative | 0 | 42 | 42 | 100.00 | NPA |
|  | Total | 11 | 42 |  |  |  |
|  |  |  |  |  |  |  |
|  | *bla*_OXA-23_ (n= 10) | URECA-LAMP | |  |  |  |
|  | WGS | Positive | Negative | Total |  |  |
|  | positive | 10 | 0 | 10 | 100.00 | PPA |
|  | Negative | 0 | 43 | 43 | 100.00 | NPA |
|  | Total | 10 | 43 |  |  |  |
|  |  |  |  |  |  |  |
|  | *bla*_KPC_ (n= 10) | URECA-LAMP | |  |  |  |
|  | WGS | Positive | Negative | Total |  |  |
|  | positive | 10 | 0 | 10 | 100.00 | PPA |
|  | Negative | 0 | 10 | 10 | 100.00 | NPA |
|  | Total | 10 | 10 |  |  |  |
|  |  |  |  |  |  |  |
|  | 16S-rRNA (n= 53) | URECA-LAMP | |  |  |  |
|  |  | Positive | Negative | Total |  |  |
|  | positive | 53 | 0 | 53 | 100.00 | PPA |
|  | Negative | 0 | 0 | 0 | N/A | NPA |
|  | Total | 53 | 0 |  |  |  |

**Supplementary Table 4 – Results of screening contrived urine samples with URECA-LAMP platform.**

| **Spiked gene (number of contrived samples)** | **Number of Positive Samples with YOLO algorithm** |
| --- | --- |
| *bla*_CTX-M-1_-group (n= 3) | 4 |
| *bla*_CTX-M-9_-group (n= 3) | 3 |
| *bla*_OXA-48_-like (n= 5)^a^ | 5 |
| *bla*_NDM_ (n= 3) | 3 |
| *bla*_VIM_ (n= 3) | 3 |
| *bla*_OXA-23_ (n= 3) | 3 |
| *bla*_KPC_ (n= 3) | 3 |
| ^a^Two strains, one carrying *bla*_CTX-M-1_-group and another carrying *bla*_CTX-M-9_-group, also carried *bla*_OXA-48-like_ genes | |

**Supplementary Table 5 – LOD results for contrived urine samples with each ESBL and carbapenemase target in URECA-LAMP panel, CFU/mL in lysate.**

|  | **Positive replicates per gene** | | | | | | |
| --- | --- | --- | --- | --- | --- | --- | --- |
| **CFU** | *bla*_CTX-M-1_-_group_ | *bla*_CTX-M-9-group*_ | *bla*_OXA-48_-like | *bla*_NDM_ | *bla*_VIM*_ | *bla*_OXA-23_ | *bla*_KPC_ |
| **10 ^7^** | - | 4/4 | 4/4 | 4/4 | 4/4 | - | 4/4 |
| **10 ^6^** | 4/4 | 4/4 | 4/4 | 4/4 | 4/4 | 4/4 | 4/4 |
| **10 ^5^** | 4/4 | 4/4 | 4/4 | 4/4 | 4/4 | 4/4 | 2/4 |
| **10 ^4^** | 4/4 | 0/4 | 4/4 | 4/4 | 0/4 | 4/4 | 0/4 |
| **10 ^3^** | 1/4 | 0/4 | 3/4 | 2/4 | 0/4 | 4/4 | 0/4 |
| **10 ^2^** | 0/4 | 0/4 | 0/4 | 1/4 | 0/4 | 3/4 | 0/4 |
| **10** | 0/4 | 0/4 | 0/4 | 0/4 | 0/4 | 0/4 | 0/4 |
| **1** | 0/4 | 0/4 | 0/4 | 0/4 | 0/4 | 0/4 | 0/4 |
| **10 ^-1^** | 0/4 | - | - | - | - | 0/4 | - |
| - = Concentration not tested  * = LOD values after centrifugation step at 14,000 RCF for 2 minutes | | | | | | |  |
